# Dr. Ferran and Cholera—Inoculation

**Published:** 1885-09

**Authors:** 


DR. FERRAN AND CHOLERA-INOCULATION.

The medical profession of the world has hoped, rather than expected, that the experiments and the claims of Dr. Ferran might prove satisfactory. On theoretical grounds it was not clear how the claims which he made should be realized. Since the disease is believed to be introduced through the alimentary canal, and one attack of it does not prevent repeated attacks, it was difficult to understand by what means he was to so impress the system, from without, as to secure immunity whenever the cause of the disease, be it comma-bacillus or anything else, found its way into the alimentary canal, and lodged in fertile soil for its development. If it be assumed that Dr. Ferran was honest in his convictions and his practice, it was heroic to have assumed the responsibility of inoculating so many human beings on the strength of theory alone, for this he seems to have done.

His expectations, his promises, appear to have failed of realization. Some who were inoculated by him have escaped death, but, if reports be true, the percentage of recoveries seems to be so small of those who have been attacked, after inoculation, as to indicate that they have been the exception rather than the rule.

By some it is claimed that the degree of confidence inspired in those who had been inoculated rendered them less liable to be attacked by the disease by diminishing the depression which follows dread of it. Even if this claim be well founded, it seems but little compensation for the apparent risk incurred by the inoculation.

Certainly, facts appear yet to be wanting to justify Dr. Ferran's claims, or further persisting in his inoculation. This seems to be the verdict of the scientists who have investigated the matter, as far as it was practicable to do so.

Thus much has been said on the assumption that Dr. Ferran has been honest—both in his belief and his practice—a claim that, in the light of the latest advices, it seems difficult to accord him.

When it is considered how great are the ravages of the disease for which no preventive is now known, and how great the sufferings, and terrible the death of those afflicted with cholera, it is inconceivable how any man, who claims to be a physician and a benefactor of mankind, should be willing to keep secret anything that he might know that could prove so great a boon to his fellow-man as a preventive or a remedy for cholera, and yet be unwilling to impart that knowledge, except for money-consideration. That he has done this is in itself sufficient to make him a self-condemned man, but if it be true as charged, and, seemingly truthfully charged, that his so-called virus is but a preparation of elaterium with which he inoculates people, and that he administers the same medicine, internally, to produce catharsis, that shall resemble the purging of cholera, what language can be found that shall be adequate to give expression to the villainy, the fiendishness, of such conduct!

